# Supplementary material for: Single-cell map of dynamic cellular microenvironment of radiation-induced intestinal injury
Source: Commun Biol. 2023 Dec 9;6:1248. doi: 10.1038/s42003-023-05645-w (PMC10710489; doi:10.1038/s42003-023-05645-w)
Supplement: Supplementary file 2 — Supplementary Information [file 42003_2023_5645_MOESM2_ESM.docx]

# Supplementary Material

**Single-Cell Map of Dynamic Cellular Microenvironment of Radiation-Induced Intestinal Injury**

Hao Lu^1†^, Hua Yan^1†^, Xiaoyu Li^1†^, Yuan Xing^1^, Yumeng Ye^1^, Siao Jiang^1,2^, Luyu Ma^1^, Jie Ping^1^, Hongyan Zuo^1^, Yanhui Hao^1^, Chao Yu^1^, Yang Li^1,3,*^, Gangqiao Zhou^1,4,*^, Yiming Lu^1,2,*^

**Affiliations:**

^1^Beijing Institute of Radiation Medicine, Beijing 100850, China;

^2^College of Life Sciences, Hebei University, Baoding City, Hebei Province, 071002, China;

^3^Academy of Life Sciences, Anhui Medical University, Hefei City, Anhui Province, 230032, China;

^4^Collaborative Innovation Center for Personalized Cancer Medicine, Center for Global Health, School of Public Health, Nanjing Medical University, Nanjing City, Jiangsu Province, 211166, China.

^†^These authors contributed equally.

^*^**Correspondence**

Dr. Yiming Lu, Department of Genetics & Integrative omics, State Key Laboratory of Proteomics, National Center for Protein Sciences, Beijing Institute of Radiation Medicine, 27 Taiping Road, Beijing, 100850, P. R. China. E-mail: ylu.phd@gmail.com; Phone & fax: 86-10-66930297.

OR

Dr. Gangqiao Zhou, Department of Genetics & Integrative omics, State Key Laboratory of Proteomics, National Center for Protein Sciences, Beijing Institute of Radiation Medicine, 27 Taiping Road, Beijing, 100850, P. R. China. E-mail: zhougq114@126.com; Phone & fax: 86-10-66931201.

OR

Dr. Yang Li, Department of Experimental Pathology, Beijing Institute of Radiation Medicine, Beijing 100850, P. R. China. E-mail: leeyoung109@hotmail.com; Phone & fax: 86-10-66930232.

**Supplementary Figures**

**
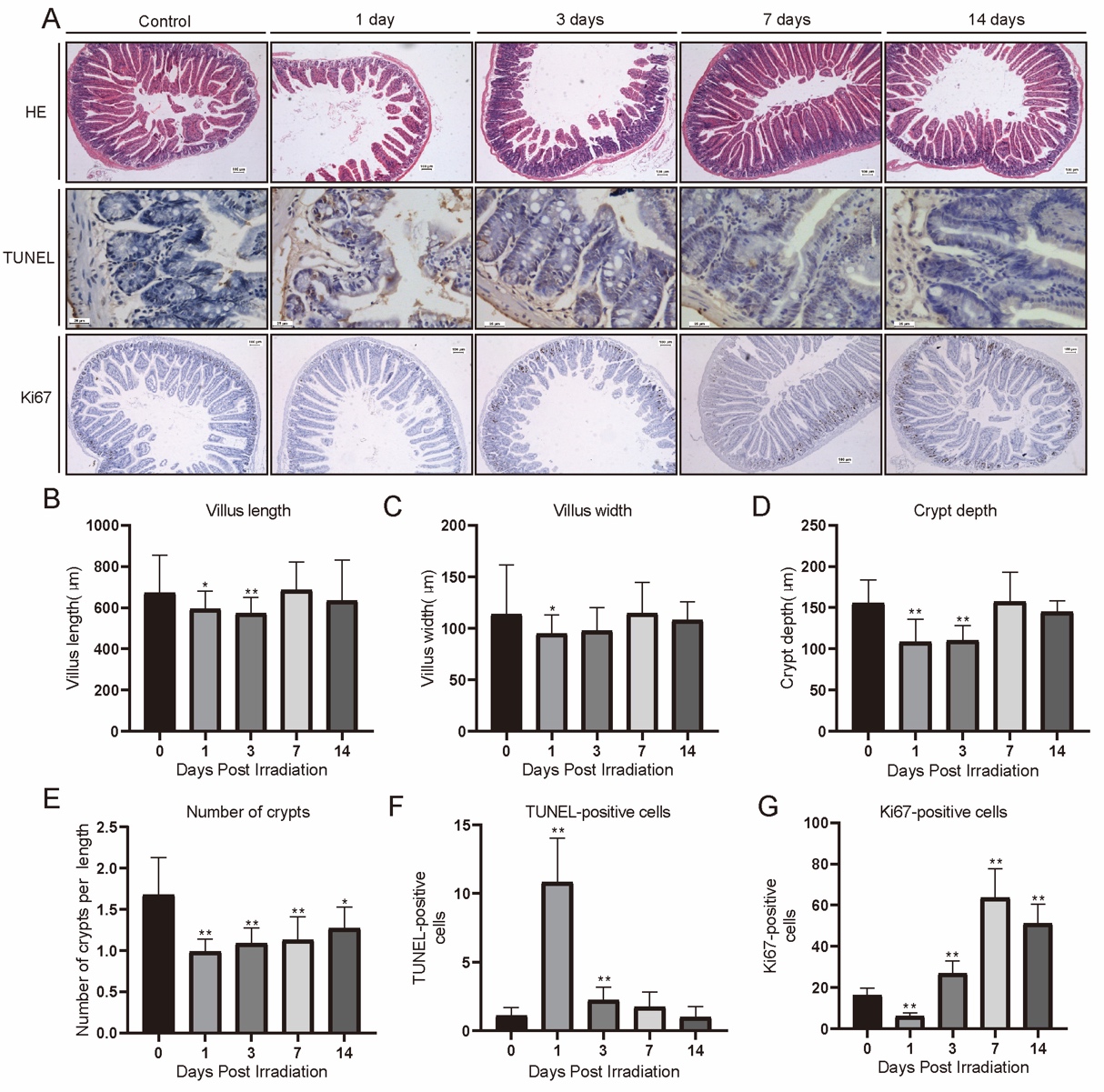
**

**Supplementary Fig. 1** **The establishment of an acute radiation enteritis model in mouse.** **a** Histopathological changes of jejunum by HE staining (scale bar = 100 μm), TUNEL staining (scale bar = 25 μm) and Ki67 staining (scale bar = 100 μm) in the control, day 1, 3, 7 and 14 groups. **b** Villus length of jejunum in each group after irradiation. **c** Villus width of jejunum in each group after irradiation. **d** Crypt depth of jejunum in each group after irradiation. **e** Number of crypts per length of jejunum in each group after irradiation. **f** Histograms of TUNEL analysis in jejunum of mouse in each group after irradiation. **g** Histograms of Ki67-positive cells analysis in jejunum of mouse in each group after irradiation. The error bars represent the standard deviation (SD).


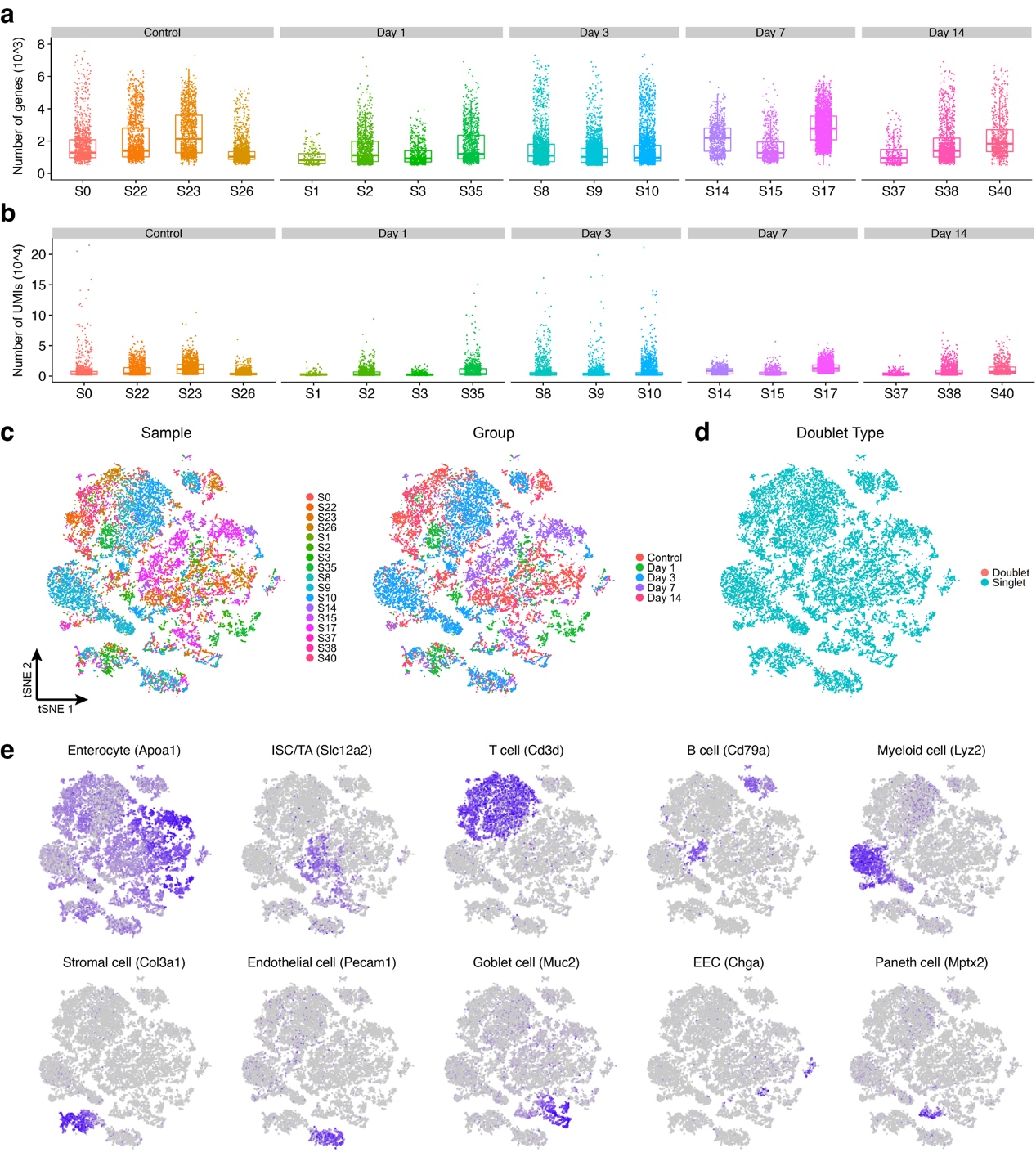


**Supplementary Fig. 2 Identifying the major intestinal epithelial cell types in scRNA-seq data.** **a**-**b** Boxplot showing the distribution of numbers of the detected genes (**a**) and UMIs (**b**) in each sample. Box center bar is the median, lower and upper hinges are the first and third quartiles, and limits of the lower and upper whiskers are the smallest and largest value no further than 1.5 times inter-quartile range extending from the first and third quartiles. Each dot represents a single cell. **c** tSNE projection of the 22,680 cells profiled, colored by sample origins (left) and experimental groups (right), respectively. **d** tSNE projection of cells colored by potential doublet types. **e** tSNE projection of cells colored by the normalized expression (gray to blue) of the selected marker genes for major cell types.


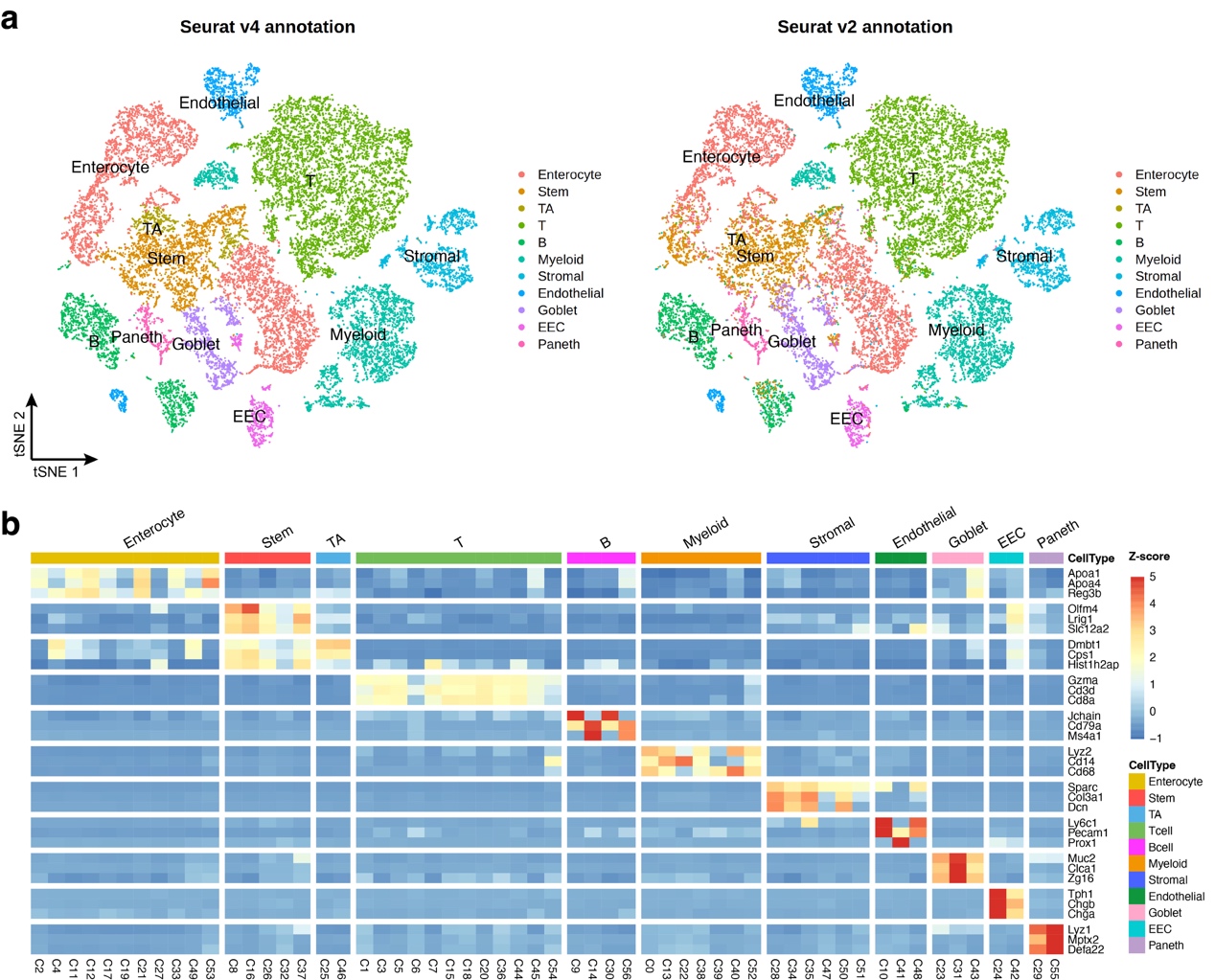


**Supplementary Fig. 3 Re-analysis of the scRNA-seq data with the updated Cell Ranger and Seurat packages.** **a** tSNE projection of cells re-analyzed with Cell Ranger v7.1.0 and Seurat v4.3.0, colored by the annotation from Seurat v4.3.0 (left) and Seurat v2.3.4 (right), respectively. **b** Heatmap displaying the z-score normalized mean expression of cell type-specific canonical marker genes across clusters identified in Seurat v4.3.0.


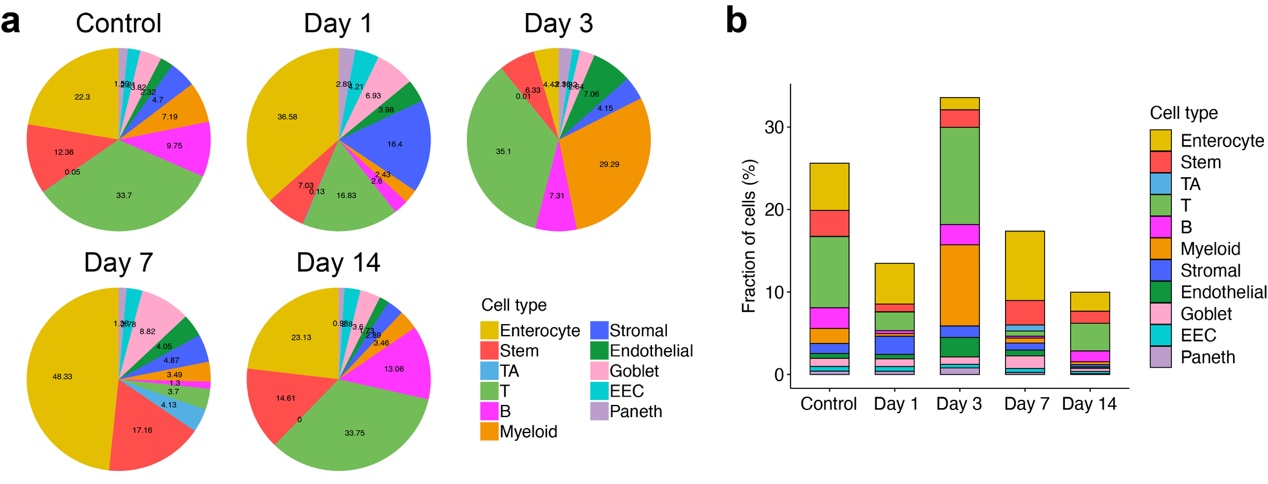


**Supplementary Fig. 4 Composition ratio of cell types at different time points. a** Pie charts showing cell type fractions in each time point. **b** Stacked bar plot showing cell type fractions by normalizing it to the total number of cells.


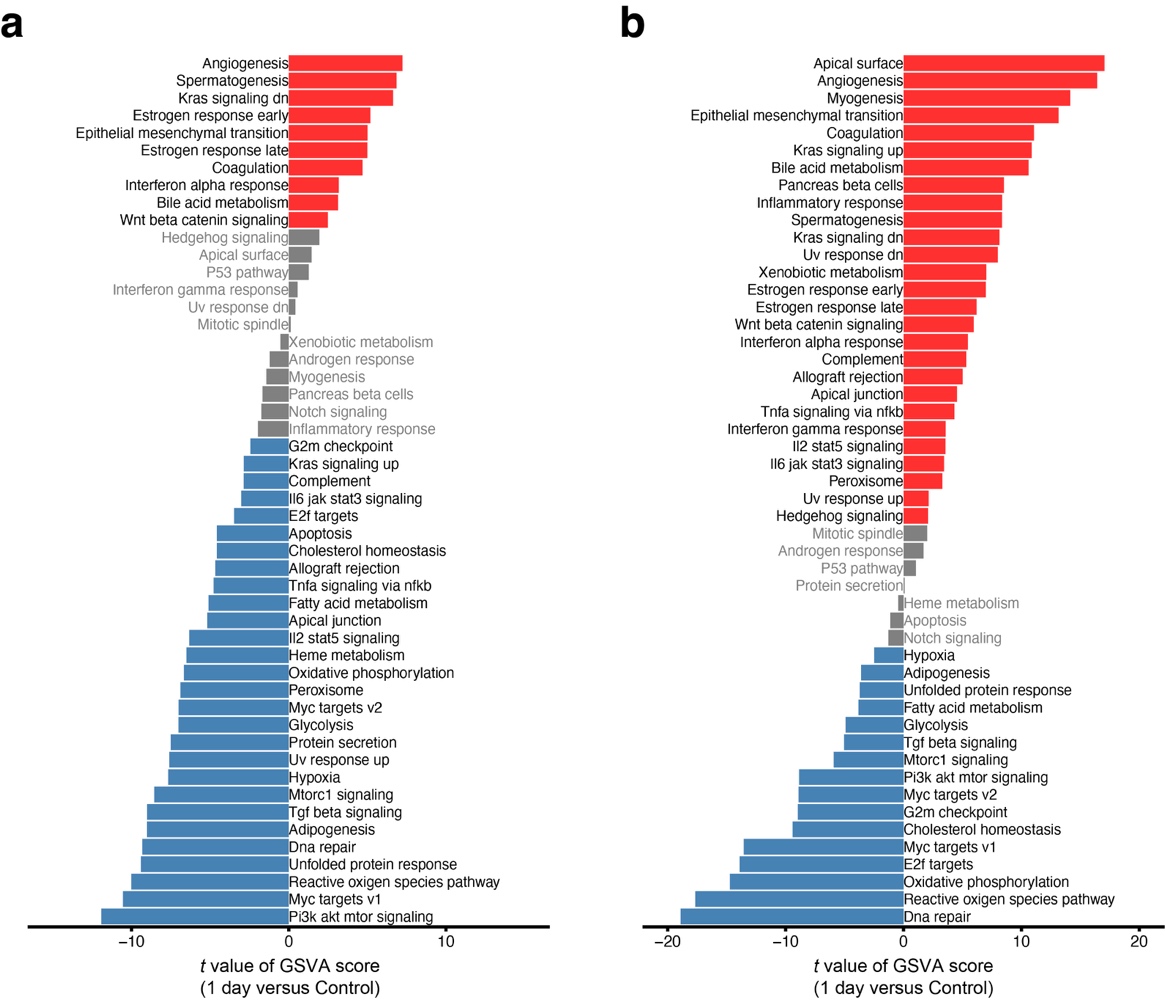


**Supplementary Fig. 5 Characteristics of *in vivo* radiosensitivities of different cell types in intestine, related to Figure 2. a** Difference of hallmark pathway activities between T cells from the control and day 1 groups. Shown are *t* values calculated in a linear modal comparing pathway scores estimated by GSVA between cells from the two groups. **b** The same as (**a**) for enterocytes from the control and day 1 groups.


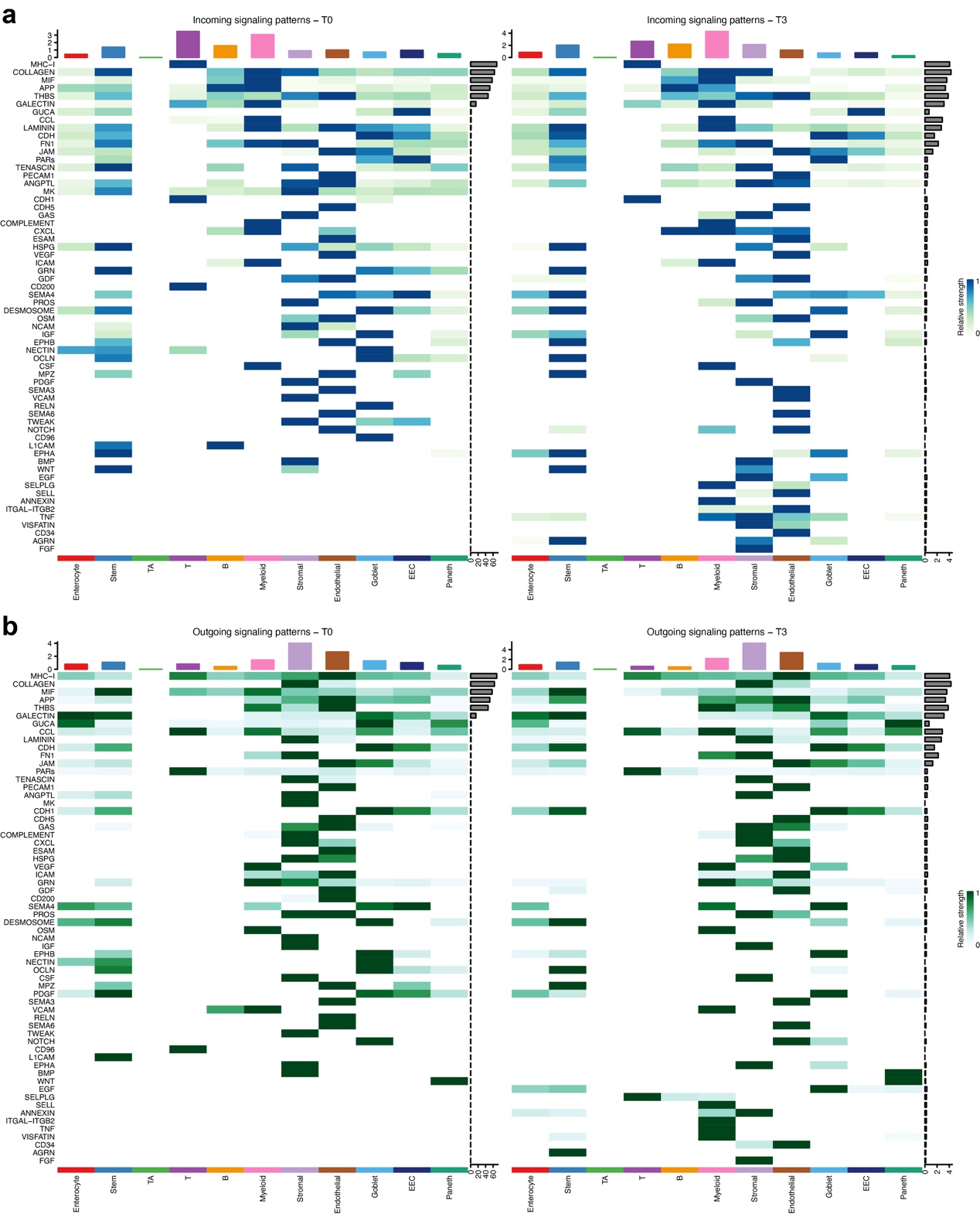


**Supplementary Fig. 6 Comparison of signaling pathways associated with each cell population between control and day 3 group. a** Heatmap showing the relative strength of incoming signaling pathways associated with each cell population in control (left) and day 3 group (right). **b** Heatmap showing the relative strength of outgoing signaling pathways associated with each cell population in control (left) and day 3 group (right).


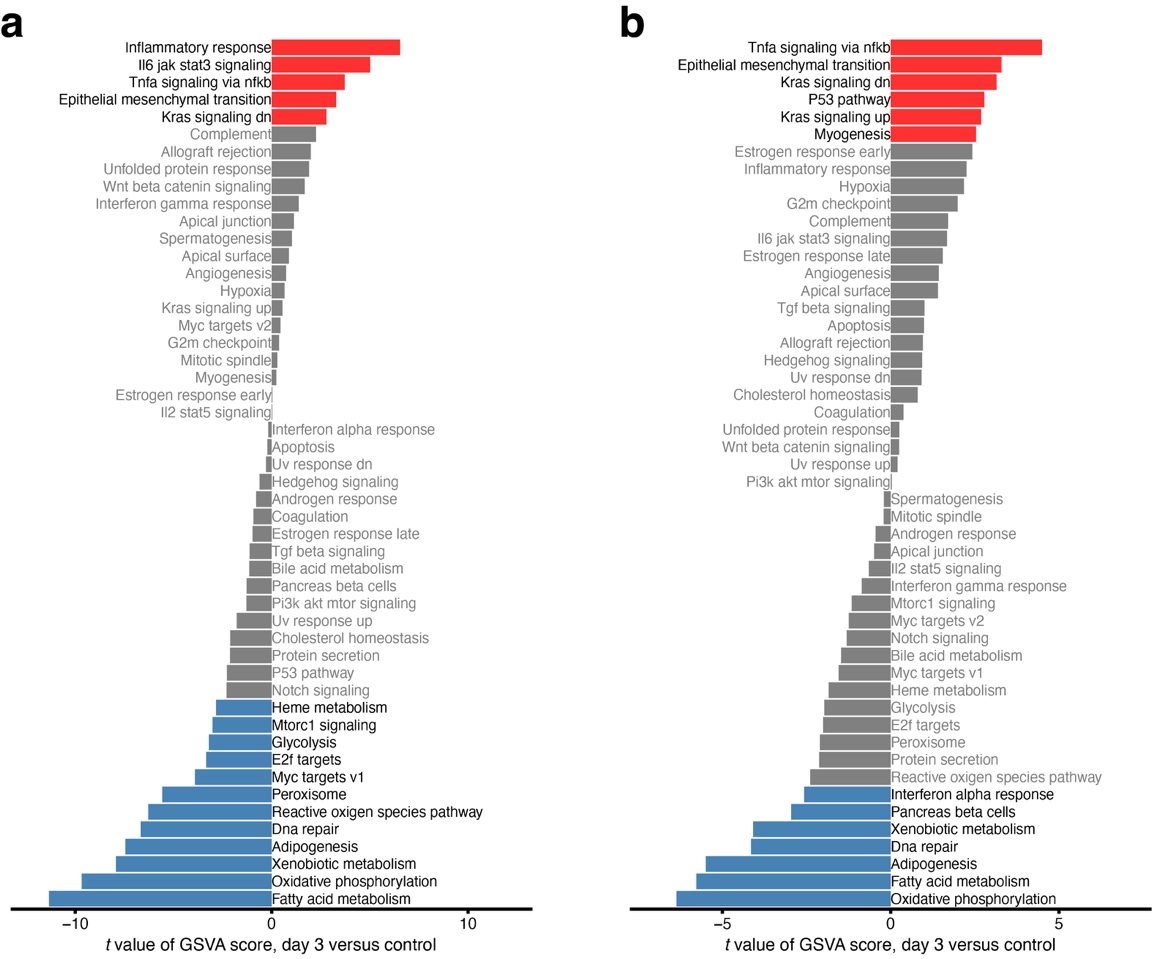


**Supplementary Fig. 7 Comparison of hallmark pathway activities in endothelial cells from day 3 and control groups. a** Difference of hallmark pathway activities between the C12-Endothelial cells from day 3 and control groups. Shown are *t* values calculated in a linear modal comparing the pathway scores estimated by gene set variation analysis (GSVA) between cells from the two groups. **b** The same as (**a**) for C25-Endothelial cells.


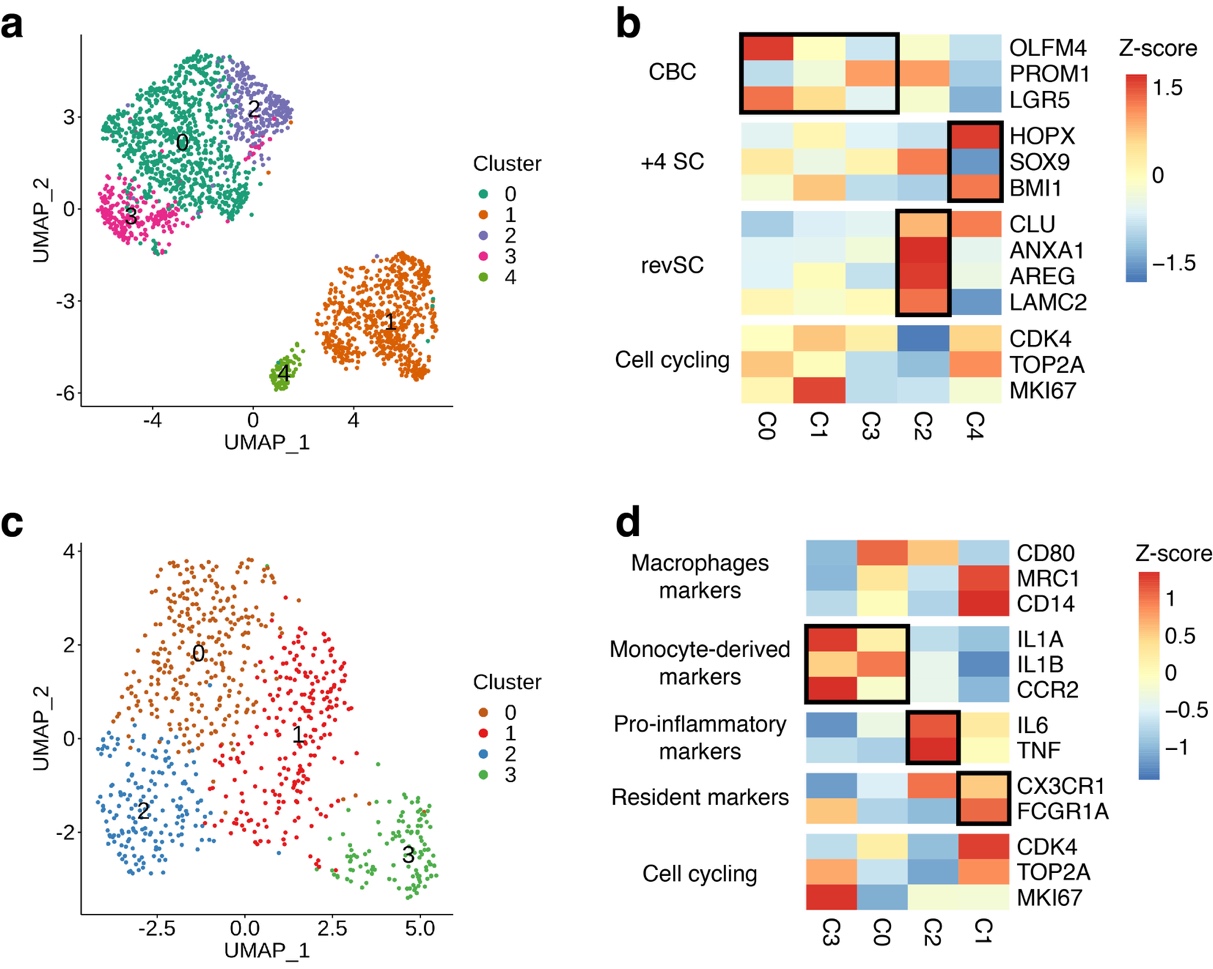


**Supplementary Fig. 8 Characterization of** **the expression profiles of stem cells and macrophages in human intestine.** The scRNA-seq data were downloaded from Fawkner-Corbett, D. et al. **a** UMAP projection of 2,045 stem cells form Fawkner-Corbett, D. et al., colored by Seurat cluster identities. **b** Heatmap of the average expression of the selected ISC function-related marker genes in the five stem cell clusters. **c** UMAP projection of 811 monocytes and macrophages form Fawkner-Corbett, D. et al., colored by Seurat cluster identities. **d** Heatmap of the average expression of the selected macrophage function-related marker genes in the four macrophage clusters.

**Supplementary Tables**

**Supplementary Table 1. Quality control statistics of each sample for single-cell RNA sequencing.**

| **Groups** | **Sample ID** | **Number of cells pasting QC** | **Mean of library size** | **Mean of genes detected** |
| --- | --- | --- | --- | --- |
| Control | S0 | 1248 | 8318 | 1757 |
|  | S22 | 1348 | 9526 | 2029 |
|  | S23 | 1306 | 13564 | 2519 |
|  | S26 | 1916 | 4353 | 1274 |
| Day 1 | S1 | 174 | 3172 | 1005 |
|  | S2 | 1134 | 5613 | 1519 |
|  | S3 | 567 | 2919 | 1168 |
|  | S35 | 1199 | 8651 | 1727 |
| Day 3 | S8 | 1956 | 6992 | 1550 |
|  | S9 | 2979 | 4366 | 1251 |
|  | S10 | 2650 | 6711 | 1513 |
| Day 7 | S14 | 776 | 8607 | 2190 |
|  | S15 | 639 | 5586 | 1617 |
|  | S17 | 2509 | 14017 | 2869 |
| Day 14 | S37 | 295 | 3896 | 1230 |
|  | S38 | 1254 | 8245 | 1862 |
|  | S40 | 730 | 11344 | 2248 |
| Total |  | 22680 | 7843 | 1778 |

Each sample represents the intestinal tissue sample from an individual mouse in corresponding group. QC, quality control.

**Supplementary Table 2. List of 67 upregulated genes after irradiation shared by T, myeloid, stem cells and enterocytes.**

| **Gene symbol** | **T cells** | | **Myeloid cells** | | **Enterocytes** | | **Stem cells** | |
| --- | --- | --- | --- | --- | --- | --- | --- | --- |
|  | **logFC** | **adjusted *P*-value** | **logFC** | **adjusted *P*-value** | **logFC** | **adjusted *P*-value** | **logFC** | **adjusted *P*-value** |
| Defa30 | 1.81 | 0.00E+00 | 1.90 | 2.36E-72 | 1.14 | 6.31E-193 | 0.98 | 1.18E-55 |
| Defa24 | 1.59 | 7.22E-128 | 0.96 | 2.64E-06 | 1.49 | 6.51E-213 | 1.38 | 1.18E-55 |
| Reg3b | 1.39 | 3.31E-129 | 1.24 | 6.21E-17 | 1.42 | 3.10E-94 | 0.61 | 7.10E-10 |
| Tff3 | 1.23 | 2.96E-106 | 0.97 | 4.81E-13 | 0.77 | 3.01E-55 | 0.46 | 3.08E-04 |
| Zg16 | 1.17 | 2.25E-98 | 0.67 | 1.95E-05 | 0.65 | 2.14E-40 | 0.27 | 6.70E-04 |
| Spink1 | 1.01 | 3.07E-68 | 1.34 | 4.23E-21 | 1.10 | 2.91E-132 | 1.11 | 3.99E-62 |
| Apoa1 | 0.93 | 1.03E-65 | 1.23 | 4.23E-21 | 0.74 | 2.96E-47 | 0.29 | 8.23E-06 |
| Reg3g | 0.73 | 8.94E-55 | 0.84 | 2.72E-11 | 0.56 | 1.77E-20 | 0.34 | 8.07E-04 |
| Spink4 | 0.72 | 2.75E-67 | 0.67 | 1.51E-09 | 0.59 | 8.44E-61 | 0.50 | 4.34E-08 |
| AY761184 | 0.68 | 2.56E-47 | 0.53 | 1.34E-04 | 0.58 | 7.38E-77 | 0.47 | 6.15E-19 |
| Fabp1 | 0.67 | 3.81E-22 | 1.16 | 1.33E-10 | 0.49 | 1.18E-17 | 0.34 | 3.64E-04 |
| Rps27l | 0.62 | 7.55E-31 | 0.62 | 1.91E-06 | 0.33 | 9.20E-30 | 0.82 | 2.33E-66 |
| Gm42418 | 0.60 | 1.10E-26 | 0.75 | 1.55E-07 | 1.06 | 7.51E-112 | 0.79 | 1.44E-25 |
| Apoa4 | 0.48 | 1.11E-17 | 0.83 | 1.36E-10 | 0.68 | 6.42E-39 | 0.19 | 3.74E-03 |
| mt-Co1 | 0.43 | 7.09E-82 | 0.49 | 9.56E-13 | 1.00 | 4.84E-142 | 0.56 | 4.49E-26 |
| Hspa1b | 0.39 | 2.37E-15 | 0.49 | 2.71E-05 | 0.07 | 4.50E-04 | 0.33 | 7.71E-13 |
| Fcgbp | 0.38 | 1.34E-33 | 0.46 | 1.86E-08 | 0.26 | 4.94E-19 | 0.15 | 3.40E-02 |
| Guca2a | 0.32 | 1.83E-31 | 0.38 | 1.65E-07 | 0.86 | 1.82E-76 | 0.35 | 4.90E-12 |
| Malat1 | 0.31 | 1.14E-16 | 0.50 | 5.69E-03 | 1.73 | 1.10E-132 | 1.28 | 2.17E-42 |
| 2200002D01Rik | 0.29 | 2.72E-06 | 0.46 | 5.10E-03 | 0.18 | 6.53E-09 | 0.60 | 5.43E-17 |
| Ccng1 | 0.28 | 1.31E-13 | 0.25 | 1.41E-02 | 0.34 | 5.90E-42 | 0.49 | 3.91E-35 |
| Defa36 | 0.28 | 1.31E-48 | 0.14 | 1.16E-04 | 0.14 | 3.76E-19 | 0.15 | 1.79E-07 |
| Anpep | 0.26 | 6.43E-16 | 0.28 | 2.09E-02 | 0.60 | 3.60E-35 | 0.21 | 8.75E-08 |
| Slc5a1 | 0.25 | 2.27E-21 | 0.48 | 1.10E-11 | 1.14 | 2.00E-114 | 0.14 | 1.62E-03 |
| Ifi27l2b | 0.24 | 2.08E-10 | 0.41 | 2.59E-06 | 0.52 | 2.29E-33 | 0.19 | 2.39E-03 |
| Arl6ip1 | 0.23 | 2.57E-04 | 0.34 | 2.78E-02 | 0.45 | 6.87E-40 | 0.25 | 1.21E-04 |
| Krt8 | 0.22 | 6.54E-06 | 0.50 | 5.06E-05 | 0.25 | 3.02E-09 | 0.61 | 1.96E-25 |
| Cck | 0.22 | 7.05E-26 | 0.26 | 2.20E-05 | 0.14 | 3.76E-17 | 0.12 | 5.24E-04 |
| Ckmt1 | 0.22 | 3.94E-07 | 0.42 | 6.10E-06 | 0.26 | 2.55E-13 | 0.15 | 3.27E-02 |
| mt-Co3 | 0.22 | 8.17E-21 | 0.35 | 8.73E-07 | 0.66 | 5.74E-106 | 0.50 | 5.69E-32 |
| Exoc4 | 0.20 | 1.88E-14 | 0.16 | 3.74E-02 | 0.05 | 7.20E-06 | 0.09 | 2.48E-04 |
| Canx | 0.19 | 1.59E-04 | 0.45 | 2.19E-03 | 0.48 | 5.93E-37 | 0.24 | 4.46E-05 |
| Gm42047 | 0.19 | 3.17E-38 | 0.14 | 1.61E-05 | 0.03 | 1.31E-07 | 0.24 | 5.26E-27 |
| Atp1b1 | 0.18 | 1.18E-09 | 0.36 | 6.75E-05 | 0.83 | 2.55E-67 | 0.20 | 4.46E-03 |
| Sct | 0.17 | 1.24E-10 | 0.28 | 2.51E-04 | 0.17 | 2.85E-11 | 0.14 | 1.39E-03 |
| Gip | 0.17 | 5.87E-09 | 0.48 | 1.38E-06 | 0.24 | 3.43E-24 | 0.25 | 4.20E-06 |
| Defa29 | 0.17 | 4.02E-18 | 0.23 | 3.89E-07 | 0.11 | 2.36E-14 | 0.07 | 3.37E-02 |
| Pls1 | 0.17 | 4.26E-05 | 0.48 | 5.48E-07 | 0.61 | 1.40E-46 | 0.19 | 1.58E-04 |
| Etnk1 | 0.17 | 3.78E-06 | 0.25 | 8.08E-03 | 0.16 | 5.04E-13 | 0.07 | 2.80E-02 |
| Slc27a4 | 0.16 | 6.50E-08 | 0.22 | 2.81E-03 | 0.54 | 1.89E-57 | 0.13 | 4.75E-05 |
| Ces2e | 0.15 | 2.69E-10 | 0.37 | 4.64E-09 | 0.86 | 3.38E-80 | 0.62 | 9.86E-43 |
| Ifit1 | 0.15 | 1.30E-09 | 0.21 | 2.92E-03 | 0.18 | 5.74E-08 | 0.05 | 2.32E-02 |
| Dcxr | 0.15 | 3.79E-07 | 0.38 | 6.63E-06 | 0.09 | 1.37E-03 | 0.21 | 2.89E-12 |
| AC149090.1 | 0.14 | 9.01E-04 | 0.23 | 1.68E-02 | 0.18 | 2.05E-17 | 0.27 | 2.92E-11 |
| Cdhr5 | 0.14 | 5.88E-06 | 0.33 | 9.01E-06 | 0.58 | 5.20E-46 | 0.15 | 5.86E-04 |
| Gls | 0.14 | 2.63E-04 | 0.37 | 2.66E-04 | 0.40 | 6.60E-41 | 0.41 | 3.68E-25 |
| Smim22 | 0.14 | 2.24E-03 | 0.46 | 8.52E-08 | 0.43 | 8.42E-34 | 0.41 | 1.86E-15 |
| Snrnp70 | 0.13 | 3.01E-02 | 0.37 | 3.86E-03 | 0.17 | 8.56E-12 | 0.38 | 8.49E-14 |
| Ggt1 | 0.13 | 1.02E-06 | 0.20 | 3.02E-03 | 0.64 | 2.28E-68 | 0.07 | 6.62E-03 |
| Slc15a1 | 0.13 | 3.47E-11 | 0.31 | 2.27E-08 | 0.77 | 2.01E-108 | 0.07 | 2.97E-05 |
| Ghrl | 0.12 | 4.57E-25 | 0.17 | 8.65E-06 | 0.10 | 3.83E-15 | 0.13 | 5.33E-05 |
| Sis | 0.12 | 1.31E-05 | 0.25 | 5.70E-05 | 0.43 | 9.02E-19 | 0.12 | 7.41E-03 |
| Slc43a2 | 0.10 | 2.05E-06 | 0.22 | 2.98E-02 | 0.68 | 9.36E-92 | 0.08 | 8.91E-04 |
| Mbl2 | 0.09 | 4.28E-13 | 0.13 | 4.48E-05 | 0.49 | 7.41E-66 | 0.07 | 1.40E-09 |
| Cdhr2 | 0.09 | 2.68E-04 | 0.29 | 4.95E-06 | 0.67 | 5.80E-82 | 0.10 | 1.70E-03 |
| Cyp4f14 | 0.08 | 3.14E-04 | 0.13 | 6.01E-03 | 0.51 | 1.63E-39 | 0.11 | 8.71E-04 |
| Gsta1 | 0.07 | 9.97E-04 | 0.17 | 7.49E-04 | 0.50 | 9.90E-28 | 0.16 | 1.90E-09 |
| Eps8l2 | 0.07 | 3.92E-03 | 0.19 | 1.78E-05 | 0.31 | 1.47E-23 | 0.20 | 3.92E-09 |
| Slc25a36 | 0.07 | 4.72E-02 | 0.18 | 1.54E-02 | 0.23 | 3.54E-21 | 0.14 | 7.67E-07 |
| Chgb | 0.06 | 1.79E-04 | 0.19 | 2.65E-04 | 0.05 | 6.65E-03 | 0.19 | 2.55E-03 |
| Selenoi | 0.06 | 7.33E-04 | 0.10 | 1.20E-02 | 0.19 | 1.57E-19 | 0.18 | 2.84E-08 |
| Myh14 | 0.06 | 1.33E-02 | 0.22 | 1.30E-05 | 0.33 | 2.71E-23 | 0.12 | 3.27E-03 |
| Mep1b | 0.06 | 1.26E-03 | 0.15 | 1.83E-03 | 0.59 | 4.05E-62 | 0.08 | 6.64E-04 |
| Tstd1 | 0.05 | 8.65E-03 | 0.23 | 3.97E-05 | 0.47 | 5.15E-37 | 0.32 | 1.02E-11 |
| Slfn5 | 0.03 | 9.02E-03 | 0.25 | 2.99E-03 | 0.05 | 1.68E-07 | 0.04 | 3.48E-04 |
| Ptprh | 0.03 | 4.29E-02 | 0.14 | 1.56E-04 | 0.25 | 1.74E-31 | 0.04 | 2.70E-02 |
| Tmigd1 | 0.03 | 4.70E-02 | 0.05 | 6.29E-03 | 0.13 | 5.78E-15 | 0.02 | 4.45E-02 |

*P* values of the differentially expressed genes were calculated based on a linear model implemented by R limma package and were then adjusted based on Benjamini-Hochberg correction using all genes in the dataset. FC, fold change.

**Supplementary Table 3. Functional annotation of the upregulated genes shared by multiple cell types after irradiation.**

| **GO ID** | **Term** | **Percentage (%)** | **P-Value** | **Genes** | **Fold Enrichment** |
| --- | --- | --- | --- | --- | --- |
| GO:0050830 | Defense response to Gram-positive bacterium | 12.5 | 1.35E-06 | REG3B, DEFA29, DEFA24, DEFA36, ZG16, REG3G, DEFA30, MBL2 | 14.11 |
| GO:0043066 | Negative regulation of apoptotic process | 10.9375 | 1.15E-02 | FABP1, CKMT1, TMIGD1, ARL6IP1, CCNG1, GHRL, HSPA1B | 3.63 |
| GO:0050829 | Defense response to Gram-negative bacterium | 9.375 | 4.03E-05 | REG3B, DEFA29, DEFA24, DEFA36, REG3G, DEFA30 | 15.40 |
| GO:0061844 | Antimicrobial humoral immune response mediated by antimicrobial peptide | 9.375 | 5.52E-05 | REG3B, DEFA29, DEFA24, DEFA36, REG3G, DEFA30 | 14.41 |
| GO:0002227 | Innate immune response in mucosa | 7.8125 | 8.16E-05 | DEFA29, DEFA24, DEFA36, APOA4, DEFA30 | 21.44 |
| GO:0019731 | Antibacterial humoral response | 6.25 | 3.25E-03 | DEFA29, DEFA24, DEFA36, DEFA30 | 13.28 |
| GO:0032532 | Regulation of microvillus length | 4.6875 | 1.72E-04 | CDHR2, CDHR5, PLS1 | 145.16 |
| GO:0070328 | Triglyceride homeostasis | 4.6875 | 4.38E-03 | APOA1, APOA4, GIP | 29.89 |
| GO:0051673 | Membrane disruption in other organism | 4.6875 | 6.62E-03 | DEFA24, DEFA36, DEFA30 | 24.19 |
| GO:0043154 | Negative regulation of cysteine-type endopeptidase activity involved in apoptotic process | 4.6875 | 1.86E-02 | FABP1, ARL6IP1, HSPA1B | 14.11 |

Functional annotation of the upregulated genes was performed using DAVID (https://david.ncifcrf.gov/). *P* values are calculated using *χ*^2^ test. Terms are sorted based on the percentages of queried gene lists in respective gene ontology (GO) terms.
