# Supplementary material for: Single-cell map of dynamic cellular microenvironment of radiation-induced intestinal injury
Source: Commun Biol. 2023 Dec 9;6:1248. doi: 10.1038/s42003-023-05645-w (PMC10710489; doi:10.1038/s42003-023-05645-w)
Supplement: Supplementary file 5 — Reporting Summary [file 42003_2023_5645_MOESM5_ESM.pdf]

nature portfolio

Corresponding author(s): Yiming Lu

Last updated by author(s): Sep 26, 2023

Reporting Summary

Nature Portfolio wishes to improve the reproducibility of the work that we publish. This form provides structure for consistency and transparency in reporting. For further information on Nature Portfolio policies, see our Editorial Policies and the Editorial Policy Checklist.

Statistics

For all statistical analyses, confirm that the following items are present in the figure legend, table legend, main text, or Methods section.

n/a	Confirmed	
☐	☒	The exact sample size (*n*) for each experimental group/condition, given as a discrete number and unit of measurement
☒	☐	A statement on whether measurements were taken from distinct samples or whether the same sample was measured repeatedly
☐	☒	The statistical test(s) used AND whether they are one- or two-sided<br>*Only common tests should be described solely by name; describe more complex techniques in the Methods section.*
☒	☐	A description of all covariates tested
☐	☒	A description of any assumptions or corrections, such as tests of normality and adjustment for multiple comparisons
☐	☒	A full description of the statistical parameters including central tendency (e.g. means) or other basic estimates (e.g. regression coefficient) AND variation (e.g. standard deviation) or associated estimates of uncertainty (e.g. confidence intervals)
☐	☒	For null hypothesis testing, the test statistic (e.g. *F*, *t*, *r*) with confidence intervals, effect sizes, degrees of freedom and *P* value noted<br>*Give P values as exact values whenever suitable.*
☒	☐	For Bayesian analysis, information on the choice of priors and Markov chain Monte Carlo settings
☒	☐	For hierarchical and complex designs, identification of the appropriate level for tests and full reporting of outcomes
☒	☐	Estimates of effect sizes (e.g. Cohen's *d*, Pearson's *r*), indicating how they were calculated

Our web collection on statistics for biologists contains articles on many of the points above.

Software and code

Policy information about availability of computer code

Data collection	Not Applicable
Data analysis	The Cell Ranger software (version 2.2.0) provided by 10x Genomics was used to align the reads from droplet-based scRNA-seq to an indexed mouse genome (mm10, NCBI Build 38); the Seurat package (version 2.3.4) was used to process the scRNA-seq expression profile; the Scrublet package (version 0.2.3) was used to identify potential doublets; the velocyto package (version 0.6) was used to perform RNA velocity analysis; the GSVA package (version 1.32.0) was used to perform gene set variation analysis; the pySCENIC package (version 0.9.11) was used to perform SCENIC analysis; the CellChat package (Version 1.6.1) was used to infer the cell-cell communication; GraphPad Prism 5 and R (version 3.6.0) were used to perform the statistical analyses.

For manuscripts utilizing custom algorithms or software that are central to the research but not yet described in published literature, software must be made available to editors and reviewers. We strongly encourage code deposition in a community repository (e.g. GitHub). See the Nature Portfolio guidelines for submitting code & software for further information.

Data

Policy information about availability of data

All manuscripts must include a data availability statement. This statement should provide the following information, where applicable:

- Accession codes, unique identifiers, or web links for publicly available datasets
- A description of any restrictions on data availability
- For clinical datasets or third party data, please ensure that the statement adheres to our policy

The scRNA-seq dataset generated in this study are available at the National Center of Biotechnology Information's Gene Expression Omniobus database under the following accession number: GSE165318.

Research involving human participants, their data, or biological material

Policy information about studies with human participants or human data. See also policy information about sex, gender (identity/presentation), and sexual orientation and race, ethnicity and racism.

Reporting on sex and gender	Not Applicable
Reporting on race, ethnicity, or other socially relevant groupings	Not Applicable
Population characteristics	Not Applicable
Recruitment	Not Applicable
Ethics oversight	Not Applicable

Note that full information on the approval of the study protocol must also be provided in the manuscript.

Field-specific reporting

Please select the one below that is the best fit for your research. If you are not sure, read the appropriate sections before making your selection.

☒ Life sciences ☐ Behavioural & social sciences ☐ Ecological, evolutionary & environmental sciences

For a reference copy of the document with all sections, see nature.com/documents/nr-reporting-summary-flat.pdf

Life sciences study design

All studies must disclose on these points even when the disclosure is negative.

Sample size	Based on a thorough review of the scientific literature, we determined that having a minimum of three samples for each experimental condition would be adequate.
Data exclusions	No data were excluded form the analyses.
Replication	We have ensured the reproducibility of our experimental findings through the implementation of biological duplication. Specifically, for each experimental condition, we maintained a minimum of three samples.
Randomization	All mice were randomly allocated to each of the experimental conditions.
Blinding	Investigators were blinded to group allocation during data collection.

Reporting for specific materials, systems and methods

We require information from authors about some types of materials, experimental systems and methods used in many studies. Here, indicate whether each material, system or method listed is relevant to your study. If you are not sure if a list item applies to your research, read the appropriate section before selecting a response.

Materials & experimental systems

n/a	Involved in the study
☐	☒ Antibodies
☒	☐ Eukaryotic cell lines
☒	☐ Palaeontology and archaeology
☐	☒ Animals and other organisms
☒	☐ Clinical data
☒	☐ Dual use research of concern
☒	☐ Plants

Methods

n/a	Involved in the study
☒	☐ ChIP-seq
☐	☒ Flow cytometry
☒	☐ MRI-based neuroimaging

Antibodies

Antibodies used	anti-Ki67(#ab16667, Abcam)
Validation	anti-Ki67(#ab16667, Abcam)<br>Suitable for: Flow Cyt(Intra), IHC-P, WB, mIHC, ICC/IF. Reacts with: Mouse, Rat, Human. References: PMID 36217678, PMID 36148577, PMID 36609277.

Animals and other research organisms

Policy information about studies involving animals; ARRIVE guidelines recommended for reporting animal research, and Sex and Gender in Research

Laboratory animals	Wild-type male C57BL/6J mice (weight of 20 ± 2g, 6-8 weeks) were purchased from Beijing Vital River Laboratory Animal Technology Co., Ltd. (Beijing, China)
Wild animals	Not Applicable
Reporting on sex	Only male mice were used in the experiments.
Field-collected samples	The study did not involve samples collected from the field.
Ethics oversight	The study was approved by the institutional review board of Beijing Institute of Radiation Medicine (protocol ID: IACUC-DWZX-2020-623).

Note that full information on the approval of the study protocol must also be provided in the manuscript.

Flow Cytometry

Plots

Confirm that:

☒ The axis labels state the marker and fluorochrome used (e.g. CD4-FITC).

☒ The axis scales are clearly visible. Include numbers along axes only for bottom left plot of group (a 'group' is an analysis of identical markers).

☒ All plots are contour plots with outliers or pseudocolor plots.

☒ A numerical value for number of cells or percentage (with statistics) is provided.

Methodology

Sample preparation	To isolate single cells, duodenums were amputated (about 5 cm beyond the pylorus) and 10 cm jejunum segments following the incision of the mice on day 0, 1, 3, 7, 14 after irradiation were washed in cold PBS, cut longitudinally into roughly 2-mm-long pieces and were isolated using the Liver Dissociation Kit (mouse) and GentleMACS Dissociator (Miltenyi Biotec, Germany) according to the manufacturer's instructions. Tissues were filtered through a 40-µm-mesh cell strainer on ice, pelleted by centrifugation at 4 °C and washed twice with the ice-cold regular medium to remove the debris.
Instrument	BD FACSCanto II flow cytometer (BD, New Jersey, USA)
Software	The cells were analyzed on a BD FACSCanto II flow cytometer (BD, New Jersey, USA) utilizing FACS Diva Software (BD)
Cell population abundance	The abundance of relevant cell populations in the post-sort fractions was analyzed using FACS Diva Software (BD)
Gating strategy	Apoptotic cells were excluded through 7-AAD staining, and CD45+ cells were established as the positivity threshold for sorting lymphocytes. CD11b and F4/80, which are typically expressed in macrophages, were set as the positivity threshold to

distinguish macrophages from other immune cells. Subsequently, we applied gating based on CD14 to identify subgroups of intestinal macrophages.

☒ Tick this box to confirm that a figure exemplifying the gating strategy is provided in the Supplementary Information.
